# Retinoblastoma-independent antiproliferative activity of novel intracellular antibodies against the E7 oncoprotein in HPV 16-positive cells

**DOI:** 10.1186/1471-2407-11-17

**Published:** 2011-01-17

**Authors:** Luisa Accardi, Maria Gabriella Donà, Anna M Mileo, Marco G Paggi, Antonio Federico, Paola Torreri, Tamara C Petrucci, Rosita Accardi, David Pim, Massimo Tommasino, Lawrence Banks, Barbara Chirullo, Colomba Giorgi

**Affiliations:** 1 Department of Infectious, Parasitic and Immunomediated Diseases, Istituto Superiore di Sanità, Rome, Italy; 2 Infective Dermatology Unit, S. Gallicano Hospital, Rome, Italy; 3 Department of Development of Therapeutic Programs, National Cancer Institute "Regina Elena", Via Elio Chianesi 53, 00144 Rome, Italy; 4 National Centre for Rare Diseases, Istituto Superiore di Sanità, Rome, Italy; 5 Department of Cellular Biology and Neurosciences, Istituto Superiore di Sanità, Rome, Italy; 6 International Agency for research and Cancer, Infectious and cancer Biology, IARC, Lyon, France; 7 International Centre for Genetic Engineering and Biotechnology, Tumor virology, ICGEB, Trieste, Italy

## Abstract

**Background:**

"High risk" Human Papillomavirus strains are the causative agents of the vast majority of carcinomas of the uterine cervix. In these tumors, the physical integration of the HPV genome is a frequent, though not invariable occurrence, but the constitutive expression of the E6 and E7 viral genes is always observed, suggesting key roles for the E6 and E7 oncoproteins in the process of malignant transformation. The "intracellular antibody" technology using recombinant antibodies in single-chain format offers the possibility of targeting a protein in its intracellular environment even at the level of definite domains thus representing a valuable strategy to "knock out" the function of specific proteins.

**Methods:**

In this study, we investigate the *in vitro *activity of two single-chain antibody fragments directed against the "high-risk" HPV 16 E7 oncoprotein, scFv 43M2 and scFv 51. These scFvs were expressed by retroviral system in different cell compartments of the HPV16-positive SiHa cells, and cell proliferation was analyzed by Colony Formation Assay and EZ4U assay. The binding of these scFvs to E7, and their possible interference with the interaction between E7 and its main target, the tumor suppressor pRb protein, were then investigated by immunoassays, PepSet™technology and Surface Plasmon Resonance.

**Results:**

The expression of the two scFvs in the nucleus and the endoplasmic reticulum of SiHa cells resulted in the selective growth inhibition of these cells. Analysis of binding showed that both scFvs bind E7 via distinct but overlapping epitopes not corresponding to the pRb binding site. Nevertheless, the binding of scFv 43M2 to E7 was inhibited by pRb in a non-competitive manner.

**Conclusions:**

Based on the overall results, the observed inhibition of HPV-positive SiHa cells proliferation could be ascribed to an interaction between scFv and E7, involving non-pRb targets. The study paves the way for the employment of specific scFvs in immunotherapeutic approaches against the HPV-associated lesions.

## Background

In recent years, recombinant antibodies have emerged as powerful tools among several different strategies for protein "knock out" [1]. Antibodies in single-chain format or single-chain variable fragments (scFvs) are extremely versatile since they can be easily delivered and opportunely manipulated. ScFvs can be either conjugated to different kinds of molecules, such as radioisotopes and toxins, or expressed as intracellular antibodies (intrabodies) in a specific intracellular compartment, where they can interfere with the function of the targeted antigen even at the level of specific domains [2].

The "intrabody" technology offers significant advantages over alternative gene therapy methods given that HPV oncogenic capacity is largely due to the interaction of viral proteins with cellular targets [3,4].

The E7 oncoprotein of the "high risk" human papillomaviruses (HPVs) is a tumor-specific antigen that plays a crucial role in virus-associated tumorigenesis, mainly by promoting cellular proliferation [5,6]. Development of therapies effective selectively at the tumor site, thus avoiding significant adverse effects on non-cancerous cells, is the main challenge in the search for new cancer treatments. Because E7 expression is restricted to the infected or transformed cells, therapeutic approaches targeting this protein offer great advantages in terms of specificity. E7 has both nuclear and cytoplasmic localization, and a pleiotropic action: it influences transcriptional and non transcriptional cell-cycle control checkpoints, subverts the expression of genes not involved in cell cycle control, and deregulates cellular energy metabolism and apoptosis [7]. More than twenty E7-binding cellular proteins have been identified so far, including transcription factors such as the members of the E2F family, but the main E7 targets are the members of the retinoblastoma family of "pocket proteins", pRb, p107 and p130. E7 binds to these proteins mostly through the strictly conserved LXCXE motif (aa LYCYE at position 22-26 in HPV16 E7) but also through the CR3 region present at its COOH-terminus [8-10]. Binding of E7 to pRb causes the release of the E2Fs and the consequent activation of genes that encode proteins promoting cell cycle progression to S phase: this association is considered the main, but not unique, cause of E7 oncogenic activity [11,12]. In fact E7 proteins from non-oncogenic genotypes, which bind to the pocket proteins with a lower affinity, are still able to deregulate cell cycle, and some E7 activities are detectable *in vivo *in pRb-null tissues [13]; furthermore, E7 promotes cell transformation by inducing an increase in protein kinase B phosphorylation [14,15]. Several oncogenic mechanisms not involving E7-pRb binding have been also reported for some HPV types [16-18].

We have previously reported the selection by "Phage Display" of three scFvs (scFv 32, 43, 51) against recombinant HPV16 E7 (16E7). One of these, scFv 43, characterized for its biological activity, showed a specific antiproliferative activity on HPV16-positive SiHa cells. This scFv was then modified by site-directed mutagenesis to generate scFv 43M2, which displayed comparable binding characteristics but improved stability [19,20].

Here we investigate the effect of scFv 43M2 and scFv 51, the most stable scFv fragments that we have generated, on cell proliferation. A significant antiproliferative effect was specifically observed in HPV16-positive SiHa cells, particularly when the scFvs were targeted to the endoplasmic reticulum, thus confirming the results obtained previously [19]. To explore the mechanisms of action underlying this activity, we looked at the binding of the two scFvs to E7, particularly in relation to the pRb binding site. Using two different deletion mutants, it was established that both scFvs bind to the N-terminal E7 sequence. The epitope mapping of the two scFvs was investigated by PepSet™assay. The possible scFvs reciprocal interference and the scFv 43M2 interference with pRb in the competitive binding to E7 were analyzed by Surface Plasmon Resonance (SPR). The results, as a whole, suggest that the epitopes recognized by the two scFvs on E7 are distinct but overlapping, and that the mechanism underlying the observed inhibition of proliferation is probably pRb-independent.

## Methods

### Plasmids, scFv fragments and cloning

The scFvs 43M2 and 51 were previously obtained by selection from the ETH-2 library of human recombinant antibodies [21]. In this library, the scFvs coding sequences are cloned in the pDN332 phagemid vector under lacZ-promoter control, in fusion with a FLAG-tag and a His-tag at their COOH-terminus. The scFv 43M2 and 51 sequences were subcloned into the Nuclear (N) and Sekdel (SD) scFvEx vectors [19], obtaining scFv 43M2N, scFv 43M2SD, scFv 51N and scFv 51SD. Briefly, the coding sequences were PCR-amplified (95°C for 1 min, 50°C for 1 min, 74°C for 1 min, 35 cycles) using the SalsekD-NotsekR2 pair of primers (restriction sites are underlined): SalsekD, 5'CGGCGTCGACCCGAGGTGCAGCTGGTGG 3'; NotsekR2, 5'CGGCGCGGCCGCTTTGATTTCCACCTTGGTCCC 3'. The PCR products were double-digested with SalI/NotI, gel-purified and ligated into scFvExpress vectors with signals for localization in the nucleus (scFvEx-N) and endoplasmic reticulum (scFvEx-SD), digested with the same enzymes. The scFvs expressed from the recombinant plasmids do not retain the original FLAG-tag and the His-tag sequences, but have acquired at their COOH-terminus a c-myc-tag, useful for detection in immunoassays.

The scFv 43M2 and 51 coding sequences, provided with specific signals for intracellular localization, were cloned into the pLNCX retroviral vector. Two constructs for each scFv, respectively targeting the nucleus (M2N, 51N) and the endoplasmic reticulum (M2SD, 51SD) were obtained.

The scFv coding sequences were PCR-amplified (95°C for 1 min, 55°C for 1 min, 74°C for 1 min, 35 cycles) using the Hind-NucDir and the Cla-NucRev pair of primers on the linearized scFvEx-N templates, and the Hind-SekdelDir and Cla-SekdelRev pair of primers on the linearized scFvEx-SD templates. The sequences of the primers used are the following (restriction sites are underlined): Hind-NucDir, 5'CCGGAAGCTTCCATGGCCGAGGTGCAGCTG 3'; Cla-NucRev, 5' CCGGATCGATCTATGCGGCCCCATTCAGATCC 3'; Hind-SekdelDir, 5' CCGGAAGCTTCCATGGGATGGAGCTGTATCATCC 3'; Cla-SekdelRev, 5' CCGGATCGATCTAGACTACAGCTCGTCCTTCTCG 3'. The PCR products were double-digested with HindIII/ClaI and ligated into the pLNCX vector digested with the same enzymes. The ligation products were used to transform *E. coli *XL1-blue cells.

The recombinant pGEX-2T vector expressing the full-length 16 E7 was kindly provided by Dr A. Venuti [22] and those expressing the 16E7 N-term, 16E7C-term and pRb as GST-fused proteins were previously described [23,24].

### Cell lines, transfection, transduction and analysis of the scFvs expression by WB and immunofluorescence staining

Two cervical epithelial tumor cell lines, the HPV16-positive SiHa (ATCC HTB 35), and the HPV-negative C33A (ATCC HTB 31) cells and the 293-derived Phoenix packaging cells (ATCC 3444) were used in this study. Phoenix cells contain the stably integrated *gag*, *pol *and *env *genes of the Moloney leukaemia virus, necessary for retrovirus assembly and replication. SiHa, C33A and Phoenix cells were maintained in Dulbecco's modified Eagle's medium (DMEM) containing 10% heat-inactivated fetal calf serum (FCS), 100 units/ml penicillin, 100 μg/ml streptomycin and 2 mM glutamine. The scFv-expressing recombinant constructs (M2N, 51N, M2SD, 51SD) and the empty pLNCX vector as a control, were used to transiently transfect the Phoenix packaging cells at 60% confluence using the JetPEI transfection reagent (Polyplus transfection) according to the manufacturer's recommendations. At 48 h post-transfection, scFv expression was monitored by WB analysis. Cell lysates in 2× SDS loading buffer were separated in 12% SDS-PAGE, blotted onto PVDF membrane and incubated with rabbit anti-c-myc mAb (clone 9E10, Sigma) followed by GAR-HRP IgG (Calbiochem, Cambridge, UK) incubation. The immunocomplexes were revealed by chemiluminescence detection. At 48 h post-transfection, the virus-containing cell culture medium from transfected cells was filtered with 0.45 μm filters and used to transduce the SiHa cells or the control C33A cells at 60% confluence. At 24 h post-transduction, cells were subcultured and plated under neomycin-selection (0.8 mg/ml). ScFv expression in transduced SiHa cells was analysed by immunofluorescence and WB. For immunofluorescence staining, performed before cell confluence, cells were fixed with 3.7% paraformaldeyde for 20 min at room temperature followed by permeabilization with 0.1% Triton X-100 in PBS for 5 min. Cells were washed with PBS and incubated for 1 h with rabbit anti-c-myc mAb, which recognizes the c-myc-tag at the scFvs COOH-terminus, followed by incubation with GAR-FITC (Cappel), both diluted 1: 50. Samples were examined using a Leitz fluorescence microscope (Germany). Cell nuclei were stained with DAPI (Invitrogen). WB was performed on SiHa cell lysates with the same procedures utilized for Phoenix cells.

### Cell proliferation and viability assays

To test cell proliferation, a colony formation assay (CFA) was performed 24 post-transduction. Cells were trypsinized, diluted 10, 100 or 1000 times, and grown for 8-15 days under G418 selection. To visualize the colonies, cells were washed in PBS, fixed and stained on the plates with crystal violet in 20% methanol.

Cell viability was tested by the EZ4U method (Biomedica Gruppe, Austria), which is based on the ability of living cells to reduce uncoloured tetrazolium salts to colored formazan derivatives. The assay was performed according to the manufacturer's instructions. Briefly, the cells were plated at decreasing dilutions on a 96-well plate and cultivated for 1-2 weeks under G418 selection. After addiction of substrate and activator the cells were incubated at 37°C for 3 h. The OD was measured at 450 and 620 nm as a reference, to correct the values for nonspecific background.

### Protein expression and purification

Full-length 16E7, two 16E7 deletion mutants, one representing the NH_2_-terminal half (16E7 N-term, aa 1-52) and the other representing the COOH-terminal half (16E7 C-term, aa 44-98) of the 16E7 protein, and the pRb protein, were expressed in *E. coli *as GST-fusion proteins. DH5α cells were transformed by the recombinant pGEX-2T vectors and purified using GST-Sepharose (Invitrogen) according to the manufacturer's instructions. The scFv expression was induced using 2 mM IPTG for 4 h at room temperature (RT) in HB2151 *E. coli *cells transformed by the recombinant pDN332 phagemid vectors. ScFv51 was extracted from the bacterial periplasm, where it is secreted by virtue of the pelB peptide leader present upstream the antibody sequence [21]. ScFv43M2 was extracted from the total bacterial lysate. Briefly, bacteria recovered from a 1-liter culture were re-suspended in 50 ml of lysis buffer (50 mM Tris-HCl, pH 7.0, 1% Triton X-100, 1 mM EDTA, 1 mM EGTA, 0.5 M NaCl) containing complete EDTA-free protease inhibitors (Roche), sonicated and incubated for 20 min on ice. The lysate was centrifuged at 12,000g for 10 min at 4°C and the supernatant processed to purify the antibody fragments. In all cases, purification was carried out by affinity chromatography on protein A-Sepharose CL-4B (Amersham Bioscience) according to the manufacturer's instructions. Purity of the proteins was evaluated by Coomassie blue staining after SDS-PAGE, and protein concentration determined by Bradford assay. The identity of the purified antibody fragments was confirmed by immunoassays using the mouse M2 mAb (2 μg/ml, Sigma, St. Louis, MO), which recognizes the FLAG-tag at the scFv COOH-terminus, followed by incubation with GAM-HRP diluted 1: 10000 (Amresco, Solon, OH). Chemiluminescence was carried out using the Super Signal West Pico Chemiluminescent Substrate (Pierce, Rockford, IL).

### Immunoassays for the determination of scFv specificity and reactivity

The full-length and deleted GST-E7 fusion proteins were immobilized on a 96-well plate (300ng/well) to perform ELISA, or separated by SDS-PAGE and transferred onto PVDF membrane (Millipore, Bedford, MA) to perform WB. For ELISA, the purified scFvs (3 μg/ml) followed by mouse M2 mAb (2 μg/ml) (Sigma, St. Louis, MO) or anti-GST mAb at 1: 1000 dilution (Santa Cruz, CA) as primary antibodies (or no antibody as a control), and GAM-HRP diluted 1: 10000 (Amresco, Solon, OH) as a secondary antibody. The immunocomplexes were revealed by Chemiluminescence as indicated above. For WB, the membrane was cut into strips (1 μg of each protein/strip) that were incubated with the same primary and secondary antibodies used in the ELISA. For the colorimetric reaction, a TMB substrate kit for peroxidase (Vector Laboratories, Inc.; Burlingame, CA) was used as a substrate.

### PepSets™ technology

PepSets™ technology (Mimotopes Pty Ltd, Clayton, Victoria, Australia) was employed for identification of the amino acids involved in the binding of E7 to the scFv 43M2 and 51 according to the method previously described [25,26]. The rod-bound peptides were assayed for their ability to physically interact with the purified scFvs. The amount of protein captured by each rod was detected via a modified immunoenzyme assay using mouse M2 mAb (1 μg/ml) and GAM-HRP at a 1: 1000 dilution. At the end of the procedure, plates were analyzed in a microtiter reader at a wavelength of 492 nm.

### Surface Plasmon Resonance by BIAcore

Surface plasmon resonance measurements were carried out using a BIAcoreX instrument (GE Healthcare) equipped with two flow-cell sensor chips. The E7 recombinant protein was immobilized to the Biacore CM5 sensor chip using conventional amine coupling: activation of the carboxymethylated dextran surface by a mixture of 0.05 M N-hydroxysuccinimide and 0.2 M N-ethyl-N0-3-(dimethylaminopropyl) carbodiimide hydrochloride. The reaction was performed by injecting E7 at a flow rate of 5 μl/min at 25°C for 7 min. Residual activated groups were blocked with 1 M ethanolamine-HCl (pH 8.5). The experiments were performed in HBS-P (10 mM Hepes pH 7.4, 0.15 M NaCl, 0.005% (v/v) surfactant P20) at 25°C. For the epitope mapping experiment the E7 surface was saturated by injection of the first scFv at a flow rate of 10 μl/min. Then, 40 μl of the second scFv were injected at 500 nM; both the scFv combinations were assayed. The response of the biomolecular interaction, expressed in arbitrary units (Response Units, RU), was monitored as a function of time (sensorgram). No nonspecific binding was revealed by the reference flow-cell. The interaction of scFv 43M2 with E7 in the presence of GST-pRb was investigated by injecting scFv 43M2 (200 nM) over E7 surface at a flow rate of 20 μl/min, in the absence and in the presence of increasing concentrations of GST-pRb (0-400 nM). The RU values of scFv 43M2 sensorgrams were measured 20 s after the injection and their variation in relation to the GST-pRB concentration was analysed with KaleidaGraph Version 4.0.

## Results

### Antiproliferative effect of the anti-E7 scFvs

We investigated whether the anti 16E7 scFv 43M2 and scFv 51, previously selected for their recognized high stability [20 and unpublished results], could specifically affect HPV16-positive SiHa cells proliferation upon intracellular expression.

**Figure 1:**
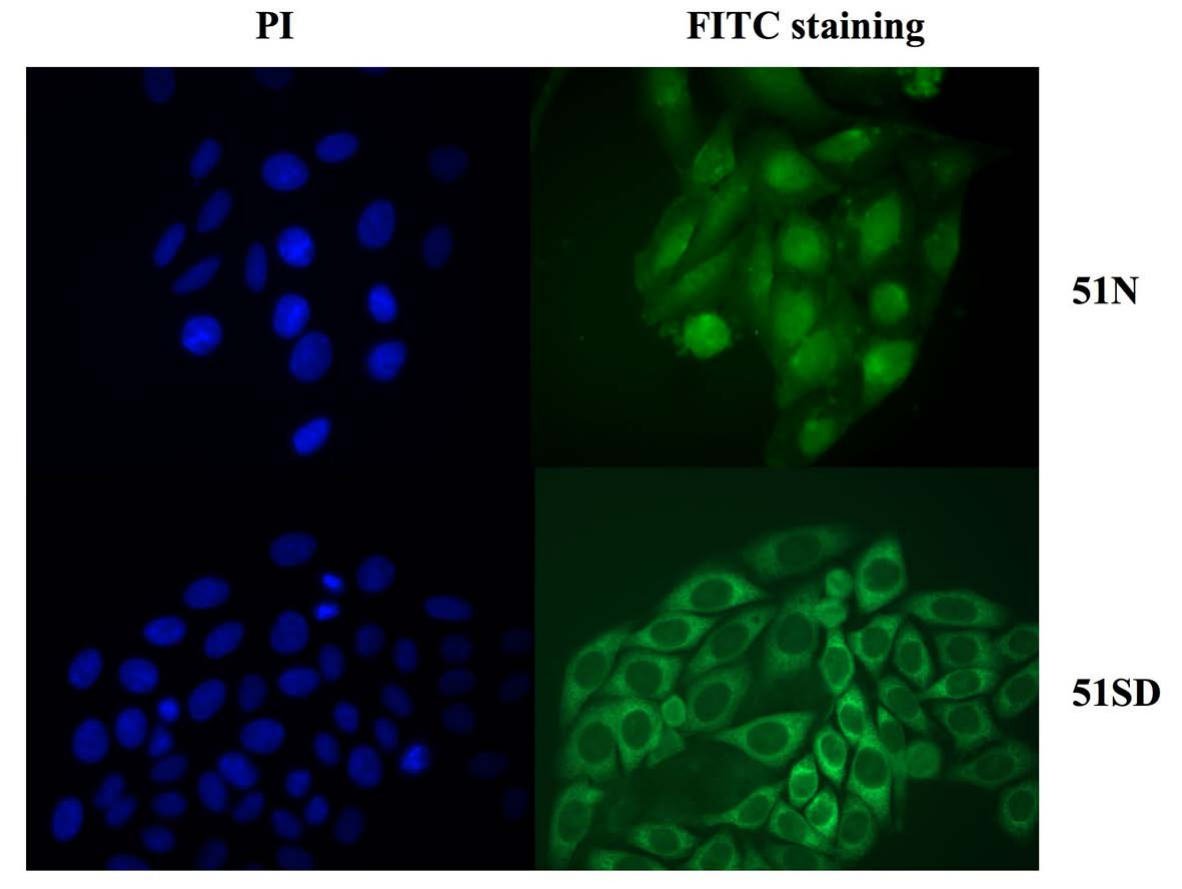
**ScFvs expression in SiHa cells by retroviral system**. SiHa cells transduced with the scFv51-expressing retroviruses carrying nuclear localization signal (51N, top panel, right) or endoplasmic reticulum localization signal (51SD, bottom panel, right) were analyzed by immunofluorescence. The transduced cells were fixed 24 h post-transduction with 4% paraformaldehyde and the scFv localization was detected by a mouse anti-myc mAb followed by an anti-mouse FITC-conjugated mAb, as a secondary antibody. The nuclei of transduced cells, shown on the left in top and bottom panels, were stained with PI.

HPV16-positive SiHa and HPV16-negative C33A cells were transduced with recombinant retroviruses expressing the scFvs in the nucleus and the endoplasmic reticulum. The localization of the expressed scFvs in cell nucleus and endoplasmic reticulum was chosen on the basis of the results obtained previously [19]. The retroviral system was utilized in order to obtain scFv expression in a higher number of cells, as compared to that observed after plasmid transfection, which was the method utilized previously. Indeed, the use of retroviral system allowed us to obtain a considerably higher percentage of scFv-expressing cells, as assessed by counting immunofluorescent scFv-positive cells. Twenty-four hours post-transduction, scFv-expressing cells were more than 30%, in comparison to 5-10% of previous experiments using transfection [19], reaching a value of 70% after a few passages under G418 selection (Figure 1).

**Figure 2:**
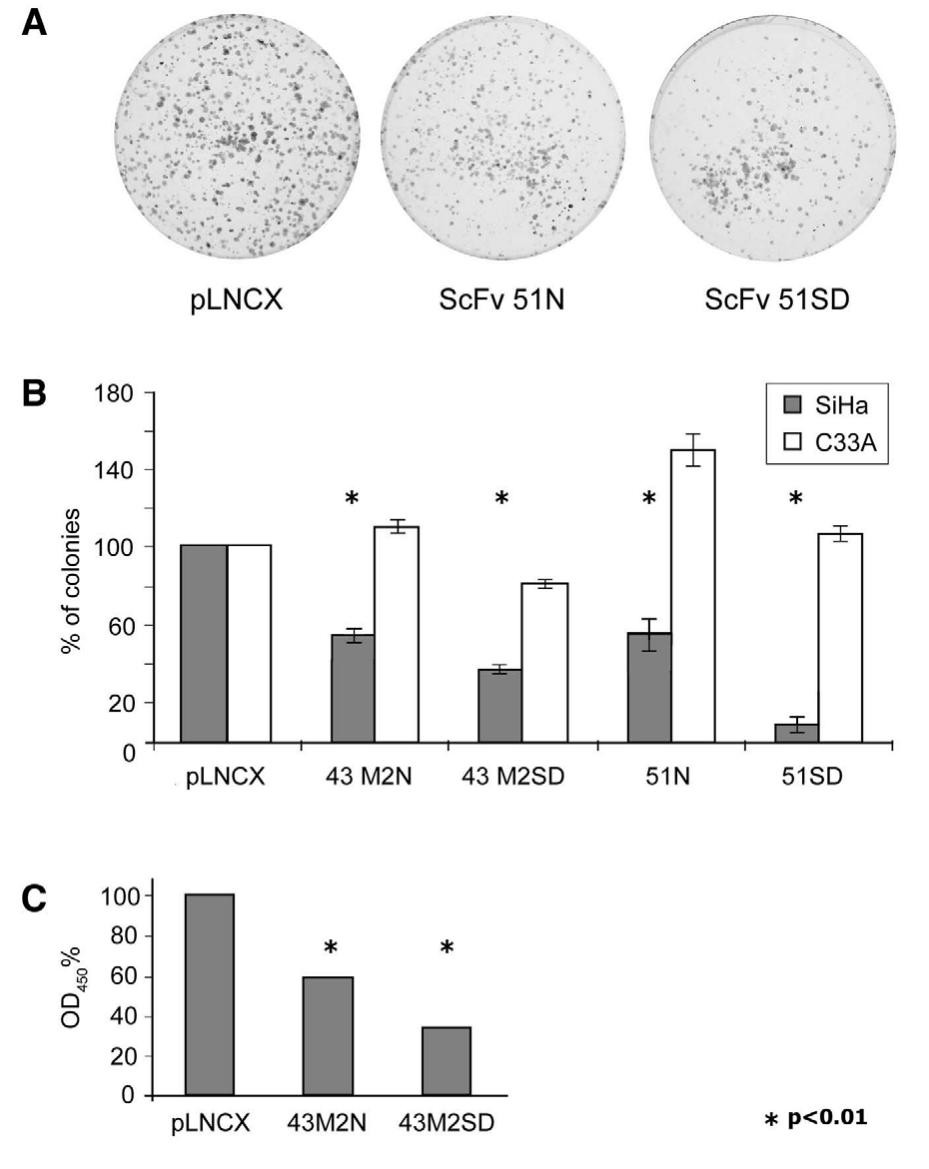
**Antiproliferative effect of the anti-E7 scFvs**. Proliferation of SiHa cells transduced with the 43M2N, 43M2SD, 51N, 51SD retroviruses and the pLNCX-retrovirus as a control was analyzed by CFA. Twenty-four hours post-transduction, the cells were G418-selected. After 2 weeks the cells were fixed with 20% methanol and stained with crystal violet and the colonies counted for each plate. The number of colonies was determined also for the C33A control cells transduced with the same retroviruses used in SiHa cells. **Panel A**: colonies obtained in a representative experiment are shown. The number of colonies in each plate was determined and expressed as a percentage of the number of cells transduced with the empty virus. **Panel B**: the histogram shows the mean percentage of colonies obtained from the recombinant retroviruses, ± standard deviation (SD) with respect to the control. The mean values ± SD were calculated from the results of three independent experiments performed using the HPV16-positive SiHa (in grey) or the HPV-negative C33A cells. **Panel C**: Analysis of cell viability by EZ4U assay. The assay is based on tetrazolium salts as indicators of cell viability. The reduction of the OD_450 _value, corrected by subtracting the OD_620 _value, is an indicator of cell death. SiHa cells transduced with 43M2N, 43M2SD and pLNCX retroviruses were grown in the wells of a 96-wells plate and the OD values were measured after 3 hours of incubation with the substrate. The percentage of the OD_450 _values of the scFv-expressing cells, corrected for the background, is reported in the figure with respect to the control cells (pLNCX). *Statistically significant data.

Cell proliferation was analyzed by colony forming assay (CFA), performed after a 14-day G418 selection (Figure 2A). The histogram in Figure 2B shows the mean values of three independent experiments. The colonies obtained from cells transduced with the M2N and M2SD retroviruses were 57% ± 3.1 and 38% ± 1.8 of those obtained from the pLNCX retrovirus-transduced cells, respectively; the colonies from the 51N- and 51SD-transduced cells were 62% ± 6.7 and 9% ± 3.8 respectively of the control cells transduced. Statistical significance of the data, with a *p *value <0.01, was assessed using the χ^2 ^test. Of note, in this assay the percentage of proliferating cells was calculated by considering the proliferation of both SiHa and C33A cells transduced with the empty retroviral vector as the reference value (100%). For this reason, the data concerning proliferating C33A cells transduced with the recombinant retroviral vectors often exceed 100%.

Cell viability of the scFv 43M2-transduced cells was investigated by the EZ4U assay, based on the capability of living cells to reduce uncolored tetrazolium salts into intensely colored formazan derivatives, assessable by an OD_450 _absorption reading. The OD_450 _values obtained for SiHa cells expressing M2N and M2SD were respectively 58% and 33% of control (Figure 2C), whereas no appreciable decrease of the OD_450 _values was observed in C33A cells, confirming the specificity of the results. These findings are in agreement with the cell proliferation values obtained by CFA. Notably, in both assays, the highest level of inhibition in cell proliferation and viability was obtained by the scFvs expressed in the endoplasmic reticulum, in agreement with the results obtained previously with a different scFv [19].

### ScFv binding to 16E7

The E7 ability to sustain proliferation is largely based on its interaction with several cellular targets the most important of which is pRb [7-18,27-29]. To possibly elucidate whether the mechanism underlying the anti-E7 scFvs antiproliferative activity was due to a competition with pRb for binding to the oncoprotein, we investigated the scFvs-binding epitopes of E7.

**Figure 3:**
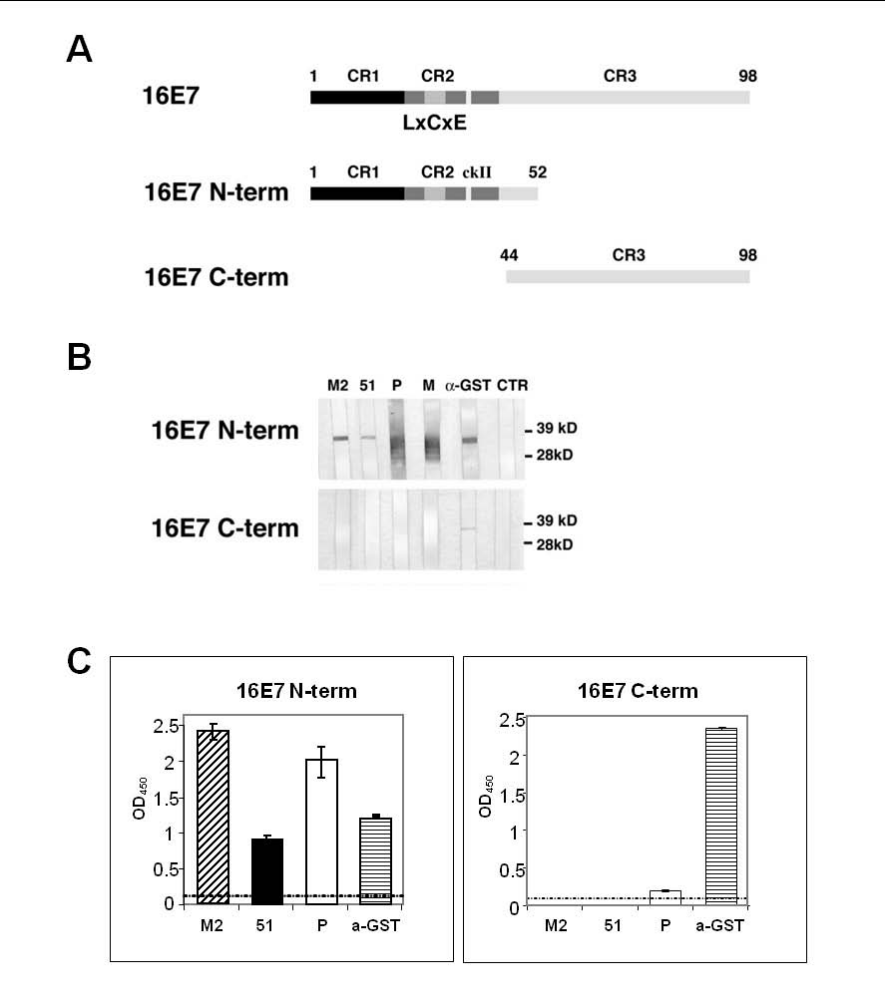
**Mapping of the scFv 43M2 and 51 epitopes on E7 protein by immunoassays**. Western Blotting and ELISA were performed using as antigens two 16E7 deletion mutants respectively representing NH_2_-term and COOH-term of the 16E7 protein. **Panel A**: schematic representation, with functional domains, of full-length 16E7 and 16E7 deletion mutants produced as GST-fusion proteins and used for the mapping of the scFvs. **Panel B**: Western blotting. Each antigen-containing strip was incubated with the purified scFvs 43M2 (M2), 51 and a mouse anti-Flag mAb. The control stripes were incubated with commercial anti-16E7 (M) and anti-GST monoclonal antibodies (mAb) in mouse, an in-house anti-16E7 polyclonal antibody (P) or no antibody (CTR). An anti-mouse peroxidase-conjugated mAb was used as a secondary antibody in all cases. The immunocomplexes were visualised by chemioluminescence. **Panel C**: ELISA. The coating was performed with the same proteins utilised for WB, and the incubation performed with the same primary and secondary antibodies, as indicated. The immune-complexes were revealed following incubation with the peroxidase substrate by reading the OD at 450 nm (OD_450_). The dashed lines represent the cut-off values for each experiment, corresponding to the OD_450 _values obtained using the anti-GST mAb.

Binding of scFv 43M2 and scFv 51 to 16E7 was analyzed by Western blotting and ELISA, using two deletion mutants representing the NH_2_-terminal half (16E7 N-term, aa 1-52) or the COOH-terminal half (16E7 C-term, aa 44-98) of 16E7 (Figure 3A). WB results (Figure 3B) showed that both scFvs were able to bind to the 16E7 NH_2_-terminal half, but not to the COOH-terminal half. These results were confirmed by ELISA (Figure 3C).

### Mapping of the E7 epitopes recognized by the scFvs

**Figure 4:**
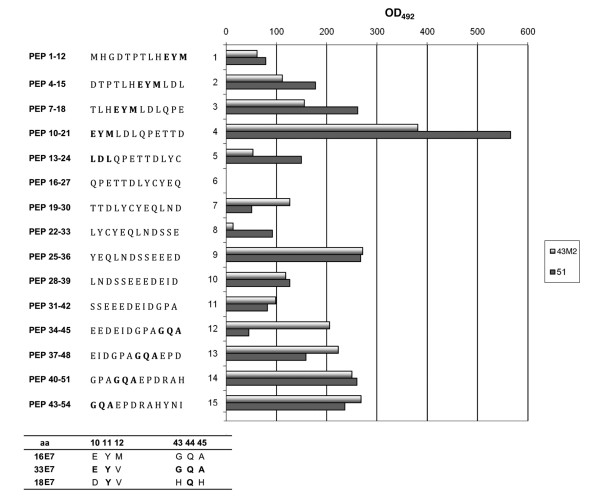
**Mapping of the scFv 43M2 and 51 epitopes on E7 protein by Pep-set™**. The HPV16 E7 amino acid sequence from 1 to 54, reproduced as 12-mers with a 9 amino acid overlap, was synthesized on 15 different polypropylene rods. The histogram represents the ability of each 12-mer oligopeptide to bind the purified scFvs as indicated in the figure. Values shown are the averages of those obtained in two different experiments with similar results. The amino acid sequences identified as probable binding epitopes are in bold. The corresponding amino acid sequences for HPV16, HPV18 and HPV33 E7 are aligned in the bottom part of the figure. The conserved residues of HPV18 and 33 in comparison with HPV16 E7 are in bold.

The linear amino acid domains of 16E7 involved in the binding to scFv 43M2 and 51 were analyzed by the PepSets™ technique. In view of the above results, oligopeptides covering the region 1-54 of the 16E7 amino acids were used. Oligopeptides were synthesized from COOH- to NH_2_-terminus on polypropylene rods as 12-mers, with a 9 amino acid-overlap. Rod-bound peptides (PEP) were then assayed by ELISA for their ability to bind to the purified scFv 43M2 and scFv 51. The average OD_492 _values of two highly reproducible experiments, are shown in Figure 4. The binding pattern of the two scFvs was similar when considering the first eleven peptides utilized, covering aa 1-42 of the E7 molecule, with a binding activity which increased from the first to the fourth peptide, reaching the maximum with PEP 10-21. Limited or null binding activity relative to PEP 16-27, 19-30 and 22-33 was observable for both scFvs. Interestingly, these are the only peptides containing the entire LYCYE pRb binding site; this result suggests it is unlikely that the two scFvs could compete with pRb for binding to E7. Furthermore, in the 34-54 aa region the two scFvs showed a different binding pattern, as scFv 43M2 but not scFv 51 bound to PEP 34-45.

The EYM (aa 10-12) and GQA (aa 43-45) sequences (both shown in bold in Figure 4), appear to play a role in the binding and could therefore represent part of the scFv binding site on E7. Nevertheless, scFv 51 scarcely bound to PEP 34-45, in which GQA is located at the COOH terminus. Furthermore, both the scFvs bound weakly to PEP 1-12, in which EYM is located at the COOH-terminus, but the scFvs binding activity towards the peptides increased with the shift of the position of the presumed epitopes towards the NH_2_-terminus. This different binding activity could be ascribed to the oligopeptide growth proceeding from COOH- to NH_2_-terminus, with the COOH- terminus bound to the rod possibly resulting in less accessibility for the physical interaction with the scFvs.

In previous experiments, we observed that scFv 43M2 was able to bind to recombinant E7 proteins of different HPV genotypes, even if with different specificities. This scFv showed a strong reactivity against the 16E7, lower reactivity against the E7 of HPV33 (33E7) and no reactivity towards the E7 of HPV18 (18E7) [19 and data not shown]. Comparison of the amino acids of 33E7 and 18E7 corresponding to positions 10-12 and 43-45 of 16E7, is shown in Figure 4 (bottom section). A high similarity between 16E7 and 33E7, with only one difference at position 12 (V instead of M) and a complete divergence between 16E7 and 18E7, with only one residue (Q) conserved at position 44, are observable. These findings support the hypothesis that these residues could play a role in the specific scFv 43M2 binding to 16E7.

### Surface Plasmon Resonance analysis of the scFv binding to E7

**Figure 5:**
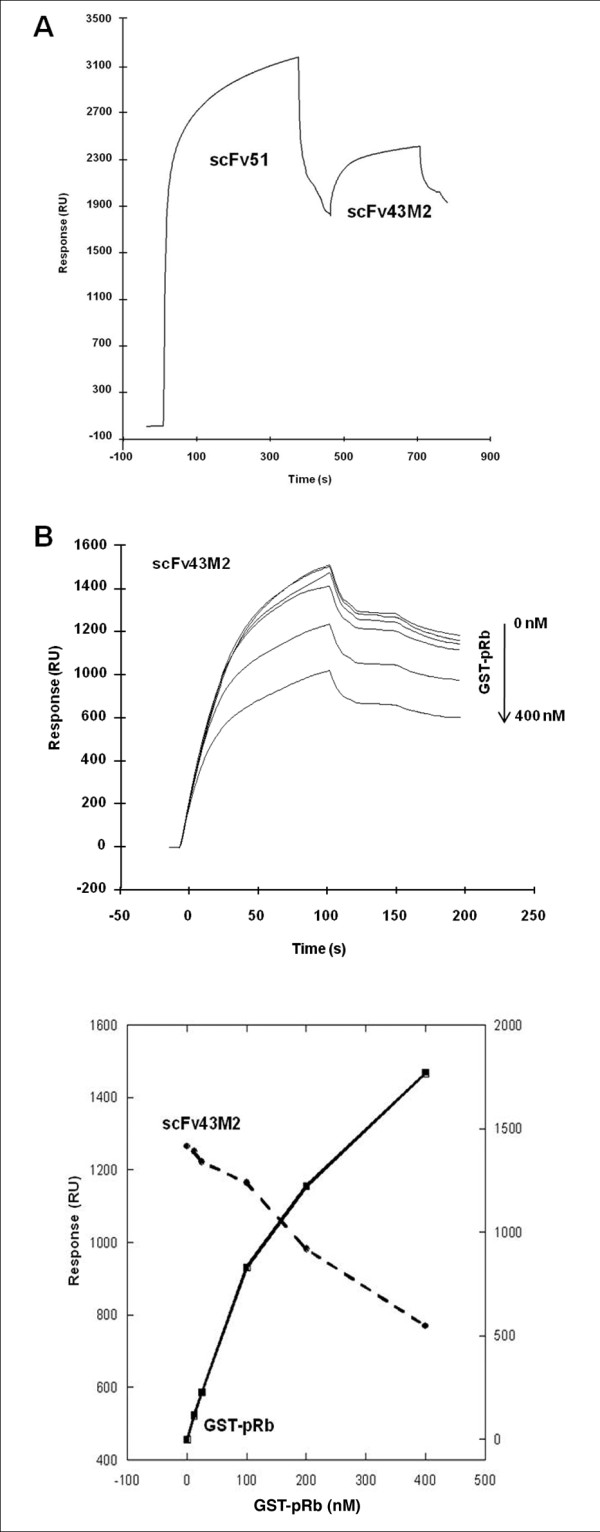
**Mapping of the scFv 43M2 and 51 epitopes on E7 protein by Surface Plasmon Resonance**. **Panel A**: the sensorgram shows the relative binding of the two scFvs to the recombinant 16E7 immobilized on the sensor chip. The scFv 51 at saturating concentration was injected followed by scFv43M2. The curves show that scFv 43M2 and 51 have distinct epitope binding sites on E7. **Panel B**, top: the scFv 43M2 binding curves in the sensorgram were obtained by injecting scFv43M2 at a constant concentration (200 nM) following the binding of GST-pRb injected over the E7 sensor chip at different concentrations in the 0-400 nM range. **Panel B**, bottom: the Response Unit (RU) values for scFv 43M2 (dashed line) as a function of GST-pRb concentrations were measured 20 s after the end of each injection. The results were elaborated with KaleidaGraph Version 4.0.

The scFv 43M2 and scFv 51 epitope specificity patterns of binding to E7 were elucidated by Surface Plasmon Resonance (SPR) using a BiacoreX-instrument. First we analyzed the binding capacity of each purified scFv to the E7 protein covalently coupled to a CM5 sensor chip. Then, to determine whether the scFvs epitopes were overlapping, each scFv was run as an analyte over the immobilized E7 after the binding of the other one. As an example, Figure 5A shows that scFv 43M2 injected at 500 nM was still able to bind to the E7 sensor chip saturated with scFv 51, suggesting the binding epitopes of the two scFvs to be distinct. The same result was obtained when the injections of the two scFvs were performed in the reverse order (not shown). Nevertheless, the RU value that expresses the binding of the second scFv injected was reduced when compared to that obtained when performing the injections in the reverse order (data not shown). This probably indicates an interference between the two scFvs due to their overlapping binding sites.

By SPR we further investigated the possibility of an overlap between the scFv 43M2 and the pRb epitopes, that could influence if not impede pRb binding to E7. Purified pRb at increasing concentrations in the 0-400 nM range was run over the E7 sensor chip followed by the injection of scFv 43M2 (200 nM) (Figure 5B). Interestingly, a non competitive inhibition was observed; in fact, with increasing bound pRb, the scFv 43M2 binding to the immobilized E7 was progressively inhibited. This binding pattern can be better appreciated in the elaboration with Kaleidograph shown in Figure 5C. Unfortunately, it was not possible to employ the GST-pRb fusion protein at the saturating concentration because pRB dissociation was hampered at concentrations higher than that used, causing chip damage.

## Discussion

Many studies have shown that the transforming activity of the "high risk" HPV E6 and E7 proteins resides in the capacity to bind to and inactivate cellular targets, including but not limited to the p53 and pRb oncosuppressor proteins, which are respectively better known targets. Inhibition of E6 and/or E7 function has been shown to inhibit the growth of HPV-positive cervical cancer cells in a number of experimental systems [30-33]. Therefore, the search for suitable inhibitors of these oncoproteins is of great relevance in terms of cancer therapy.

We have previously demonstrated the inhibition of HPV16-positive SiHa cell proliferation by an anti-16E7 scFv [19]. In this study we present the anti-proliferative activity of two other anti-16E7 scFvs. We found that the two scFvs negatively affect cell proliferation when expressed in nucleus and ER of SiHa cells, and have no effect in the HPV-negative C33A cells, showing the specificity of their activity.

The most efficient inhibition of cell proliferation was obtained when the scFvs were expressed in the endoplasmic reticulum; this is probably ascribable to the high stability of antibody fragments when they are retained in this compartment, and it has already been discussed elsewhere [19]. E7, previously regarded as primarily a nuclear protein, has been found both inside and outside the cell nucleus, and recent findings support its functional relevance in non-nuclear compartments. The presence of E7 in the ER and in the cytoplasm has been suggested and demonstrated, as well as the E7 interaction with a number of cytoplasmic proteins [33-35]. As an example, specific association of the 16E7 with γ-tubulin has been shown and suggested to contribute to E7-induced centrosome overduplication, which is considered one of the mechanisms underlying the E7 ability to deregulate cell proliferation [27]. Therefore, even though the mechanisms underlying the anti-E7 scFvs antiproliferative effect are not yet fully understood, it is very likely that these molecules exert their activity by functioning outside the nucleus.

We then addressed the molecular mechanisms underlying the scFvs anti-proliferative activity by analyzing the scFvs-binding epitopes of E7. Since the relationship between the E7 activity and its interaction with pRb is well established, we focused on the possible interaction of the scFvs with the pRb binding site. In this regard, we wondered whether the observed inhibition of cell proliferation by the scFvs could be due to competition with pRb for binding to E7. By immunoassays we showed the epitopes of both the scFvs to map to the E7 NH_2_-terminal half (aa 1-52). Analysis by PepSet™technology and SPR permitted us to investigate different aspects of the scFv binding. These different methodologies allowed us to analyze the binding to linear epitopes (PepSet™), as well as the binding between two molecules in their steric configuration (SPR).

By PepSet™analysis, it was shown that both the scFvs bound predominantly to PEP 10-21 and with low or null efficiency to the peptides containing the main pRb binding site. Two sequences each consisting of three amino acids were identified as possibly belonging to the E7-binding epitopes of both the scFvs. Interestingly, when observing the binding activity in relation to the position of such epitopes in the respective oligopeptides, localization at the COOH-terminus appeared to negatively influence the binding. Therefore, the position of a specific domain in a given context seems to be a key aspect of the binding even when considering a linear arrangement. However, some differences between the two scFvs in terms of E7 binding were highlighted by this analysis, supporting the argument that the epitopes of the two scFvs are at least partially distinct.

The difference between the epitopes of scFv 43M2 and scFv 51 on E7 binding was confirmed by SPR. In this experiment we observed that each scFv could bind to the E7 immobilized on the chip even when the other scFv was still bound. Furthermore, we observed a non-competitive inhibition by pRB, of the binding between scFv 43M2 and E7, where increasing concentrations of pRb were able to proportionally reduce the binding of 43M2 to the E7 immobilized on the chip. This finding is only apparently in contrast with the PepSet™results suggesting that the LYCYE motif (pRb binding site) is not directly involved in the scFv binding. In fact, the adjacent amino acids outside the LYCYE pocket could contribute to the binding when E7 is partially folded in its three-dimensional structure (see SPR analysis). However, we cannot completely exclude the possibility that the inhibition of scFv 43M2 binding by pRb is simply due to steric hindrance of the recombinant GSTpRb molecule, which is larger than the scFv molecule (130 kDa versus 27 kDa). This potential drawback cannot be circumvented in this experimental model.

Of note, in pRb-null Saos-2 cells transfected with the scFv 43M2- and pRb-expressing plasmids, we did not observe a rescue of pRb level when expressing scFv 43M2 in the ER (data not shown), supporting the hypothesis that the proliferation inhibition by scFv 43M2 is not pRb-dependent.

Our results on the analysis of scFvs binding to E7 support the possibility that interaction with regions other than the E7 LYCYE motif underlies the scFvs activity. We cannot exclude the possibility that interaction with other cellular components could be involved in this inhibition. Indeed, pRb tumor suppressor and the other pocket proteins are expressed in different relative amounts according to the differentiation state of the tissue [8,9]. It has also been observed that the E7 NH_2_-terminal region upstream of the LXCXE pocket, is involved in the degradation of the pRb family of proteins, even though it is not involved in their binding to E7 [36]. A number of studies have described non-pRb E7 targets which are important in cervical dysplasia or invasive cancer etiology [13-18,27] as well as models by which E7 destabilizes pRb indirectly through other cellular components [28,29].

Furthermore, the E7 NH_2_-terminus has been recently described as an intrinsically disordered or "natively unfolded" domain, able to adopt different conformations depending on pH and other cellular environmental conditions, thereby conferring conformational plasticity to the oncoprotein [37,38]. The plasticity derives directly from the capability of a α-helix to undergo β-sheet transitions, thus modifying the local structure to optimize the interaction with other proteins. In this regard, it can be hypothesized that, by virtue of their capacity to bind to the E7 NH_2_-terminus, these scFvs can counteract the conformational shift thereby preventing interaction with other cellular targets.

## Conclusions

The anti-proliferative capacity of the two anti-E7 scFvs with different epitope specificity, reported in this study, might contribute to paving the way for the design of therapeutic molecules against HPV-associated cancers, an approach which should not be by any means undervalued in the near future. Considering the epitope mapping results together, we can draw the conclusion that both the scFv 43M2 and scFv 51 bind to a region mapping in the 16E7 NH_2_-terminal half but not corresponding to the pRb binding site; however, other regions located upstream and downstream the pRb binding site, and probably involved in determining the secondary structure of E7, seem to contribute to this binding, and may well interfere with the E7 binding to pRb. When expressed intracellularly, the two scFvs may work by inhibiting various activities of the E7 protein. It is also possible that their contribution is different in different kinds of tumors and experimental systems. Elucidation of the targets involved in this inhibition and further investigations on the mechanism of the functioning of the scFvs are definitely required. Importantly, the possibility of expressing the scFvs in different cellular compartments offers the chance to modulate their action according to the target localization. ScFvs with different binding specificity could be employed either independently, to discriminate the different functions of E7 thus gaining an insight into the mechanism of E7-mediated deregulation of cell proliferation, or in combination, to enhance their anti-E7 activity. Finally, as stability of the recombinant antibodies is an important issue for their bioavailability, the possible use of scFvs-expressing constructs would represent an advantage in terms of delivery of therapeutic molecules in clinics.

## Abbreviations

CFA: colony-formation assay; GST: glutathione-s-transferase; GAM-HRP: goat anti-mouse antibody horseradish peroxidase-conjugated; GAR-HRP: goat anti-rabbit antibody horseradish peroxidase-conjugated; IPTG: isopropyl thio-β-D-thiogalactoside; RAM-HRP: rabbit anti-mouse antibody horseradish peroxidase-conjugated; GAR-FITC: goat anti-rabbit IgG FITC-labelled; Ala: alanine; Asp: asparagine; Cys: cysteine; Gln: glutamine; Glu: glutamate; His: histidine; Leu: leucine; Pro: proline; VH: heavy chain variable region; His-tag: Histidine-tag; HPV: Human papillomaviruses; mAb: monoclonal antibody; NLS: nucleus localization signal; OD: optical density; PVDF: polyvinylidene difluoride; PI: propidium iodide; pRb: retinoblastoma protein; scFvE: scFvExpress; scFv: single-chain variable fragment; SPR: Surface Plasmon Resonance; WB: Western blot analysis.

## Competing interests

The authors declare that they have no competing interests.

## Authors' contributions

LA designed and coordinated the study, carried out the molecular studies, performed the retroviral transductions and the experiments of cell proliferation, and drafted the manuscript. MGD set up the retroviral transduction system. AMM and MGP designed the PepSet experiments and critically revised the manuscript; AF performed the PepSet experiments; PT participated in the design and performed the SPR experiments. TCP designed the SPR experiments and critically revised the manuscript. RA participated in setting up the retroviral transduction system. DP participated in molecular studies and revised the manuscript, MT and LB participated in the study design and critically revised the manuscript. BC carried out the immunoassays. CG participated in the study design and critically revised the manuscript. All authors read and approved the final manuscript.

## Pre-publication history

The pre-publication history for this paper can be accessed here:

http://www.biomedcentral.com/1471-2407/11/17/prepub
